# Effect of deep litter housing and fermented feed on carcass characteristics and meat quality of crossbred Hampshire pigs

**DOI:** 10.14202/vetworld.2015.881-887

**Published:** 2015-07-16

**Authors:** M. Rahman, J. R. Bora, A. K. Sarma, R. Roychoudhury, A. Borgohain

**Affiliations:** Department of Livestock Production and Management, College of Veterinary Science, Assam Agricultural University, Khanapara, Guwahati-781 022, Assam, India

**Keywords:** fermented feed, deep litter housing, crossbred Hampshire pig, carcass characteristics, meat quality

## Abstract

**Aim:**

The objective of the study was to evaluate the effect of deep litter housing and fermented feed on carcass characteristics and meat quality of crossbred Hampshire pigs.

**Materials and Methods:**

Forty-eight weaned crossbred Hampshire piglets of 2 months age (24 males and 24 females) were selected for the experiment. The piglets were randomly assigned into 4 homogenous experimental groups with 6 males and 6 females each: E_1_; reared on a conventional housing and fed with a fermented diet, E_2_; reared on a deep litter housing system and fed with a fermented diet, E_3_; reared on a deep litter housing system and fed with a conventional diet and C; reared on a conventional housing system and fed with a conventional diet. The study was continued up to 32 weeks of age and at the end of this period, 6 animals (3 males and 3 females) from each experimental group were slaughtered to evaluate carcass traits and meat quality characteristics.

**Results:**

Pre-slaughter weight, hot carcass weight, and dressing percentage were significantly (p<0.01) affected by feeding fermented diet and deep litter housing while carcass traits, i.e., carcass length, backfat thickness, and loin eye area were not affected. The edible offal; liver and heart weight (p<0.05) differed significantly while kidney weight showed no difference. The inedible offal; head weight (p<0.01) and lung weight revealed a significant difference (p<0.05) while spleen and stomach weight showed no difference among the experimental groups. The wholesale cuts and meat: bone ratio of pigs also differed significantly among the groups. Morphometry of small and large intestine also showed a significant difference. Chemical composition of pork *viz*., moisture and total ash content was influenced by the treatment, while crude protein and ether extract content were not affected. Mineral composition of pork also showed no significant difference. Color characteristics of *Longissimus dorsi* muscle showed a significant difference in L* and a* value while parameter b* was not affected. The tenderness of meat showed significant difference among the groups (p<0.01).

**Conclusion:**

Crossbred Hampshire pigs being reared on fermented feed and deep litter housing could produce highlygraded carcass and improvement in meat quality.

## Introduction

Conventional pig production systems are generally thought to be associated with poor animal welfare that results in meat quality deterioration [1]. It is generally accepted that environmental enrichment with substrates improves the welfare of growing pigs [2]. Research conducted elsewhere have shown that average daily body weight gain, feed conversion ratio, and survival rate of pigs raised in deeplitter housing system to be equal or superior to those raised in conventional concrete floor pig houses [3,4]. There are also benefits for animal welfare by providing a more comfortable lying surface, result in less joint lesions in deep litter system. In this system, pigs get a constant source of manipulable material to exhibit rooting and other natural behaviors [5]. Thus, it is very important to implement pig production systems that satisfy consumer and citizen demands for lower environmental impact, improved animal welfare, and meat quality.

There is a focus on feeding fermented diet to the pigs as improvement in growth performance through better assimilation of nutrients in the gut and maintenance of gastrointestinal health through improvement in gut microbial ecology have been reported by several researchers [6]. The improved growth performance through feeding of fermented diet to pigs has also been associated with improved carcass characteristics [7]. Besides, it was also reported that tenderness of the pork was increased when pigs were fed with fermented feed [8]. Color parameters of the loin muscle appeared to be improved after feeding fermented feed to the pigs [9].

An experiment was therefore conducted to study the carcass traits and meat quality characteristics of crossbred Hampshire pigs fed with a fermented diet and reared on a deep litter housing system.

## Materials and Methods

### Ethical approval

The experiment was approved by the Institutional Animal Ethical Committee, College of Veterinary Science, Khanapara, Assam Agricultural University, Guwahati – 781022(Approval No: 770/ac/CPCSEA/FVSc/AAU/IAEC/11-12/124).

### Study area

The experiment was conducted in the pig farm of the National Agricultural Innovation Project (Component-2), College of Veterinary Science, Assam Agricultural University, Khanapara, Guwahati-22. Forty-eight weaned crossbred (Hampshire X Assam local) 2-month-old healthy piglets were used in this experiment. The animals were divided randomly into 4 homogenous groups with 6 males and 6 females each: E_1_; reared on a conventional housing and fed with a fermented diet, E_2_; reared on a deep litter housing system and fed with a fermented diet, E_3_; reared on a deep litter housing system and fed with a conventional diet and C; reared on a conventional housing system and fed with a conventional diet.

### Housing of animal and feed fermentation

In the conventional system, the pigs were kept on a cement concrete floor and in the deep litter housing system, pigs were reared on fermented bedding consisted of sawdust (40%), paddy husk (20%), dry soil (20%), charcoal (10%), dried tree leaves (10%), extract of fermented bamboo shoot, black salt, water (less than 30%), and active culture of *Lactobacillus brevis*. Conventional feed prepared with various feed ingredients and the parts used are presented in Table-1. For fermentation of feed *Lactobacillus plantarum* (221) strain was used @ 6 log_10_cfu/g of feed where feed and water mixed at 2:1 ratio. Proximate composition of conventional and fermented feed are depicted in Table-2.

**Table-1:** Parts of ingredients used in the preparation of feed.

Ingredients	Grower	Finisher
Maize	50	55
Wheat bran	22	22
Groundnut cake	15	10
Soyabean meal	10	10
Mineral mixture	2.5	2.5
Common salt	0.5	0.5
Total	100.00	100.00
Additives:		
Vitamin @ 10g/quintal		
Lysine @ 30g/quintal		
Methionine @ 15g/quintal		

**Table-2:** Proximate composition of conventional and fermented feed.

Feed	Proximate composition (%)				

	Moisture	Crude protein	Crude fiber	Ether extract	Total ash
Grower					
Conventional	10.78±0.035	18.15±0.22	4.24±0.04	4.08±0.025	5.31±0.085
Fermented	55.52±1.14	19.90±0.22	4.10±0.05	4.17±0.09	4.96±0.06
Finisher					
Conventional	10.82±0.10	15.96±0.215	5.54±0.20	4.39±0.10	5.26±0.04
Fermented	55.98±0.43	17.49±0.435	5.17±0.12	4.49±0.30	4.63±0.25

### Data collection

At the end of the experiment (32 weeks) 6 pigs (3 males and 3 females) from each experimental group were slaughtered to study the carcass traits and meat quality characteristics. Before, slaughter pigs were overnight starved and water was offered *ad libitum*. Pre-slaughter weight (kg) of pigs was measured by a digital platform balance. Humane (painless) method of slaughter was followed (the pigs were electrically stunned before bleeding). Hot carcass weight (kg) was also recorded in a track balance prior to chilling and dressing percentage was estimated.

The edible offal *viz*., heart, liver, and kidney were also weighed. At the same time, the weight (kg) of inedible offal and parts *viz*., lungs, spleen, alimentary tract, head were recorded immediately after evisceration. Finally, the length (m) and diameter (mm) of small and large intestine were measured by using a measuring tape after removal of the intestinal content, while their weights (kg) were recorded in a digital balance.

Carcass length was measured in centimeter. The back fat thickness was measured with a metallic tap scale at the level of the first rib, last rib, and last lumbar vertebrae. The average of the 3 measurements was calculated as back fat thickness and expressed in centimeter. The width of loin eye area (circumference of *Longissimus dorsi* muscle in between the 10^th^ and 11^th^ rib) was measured by a tracing paper by placing it against the cut surface of the eye muscle. The respective area (cm^2^) was measured by using a compensating polar planimeter.

The weight (kg) of wholesale cuts; ham, bacon, loin, boston butt, picnic, and jowl was measured in a digital balance. The chilled meat was separated from the carcass and weight of the meat and bone was estimated to calculate the meat: bone ratio.

Chemical composition *viz*., moisture, crude protein, ether extract, and total ash content of *L. dorsi* muscle were analyzed following the procedure described by AOAC [10]. Mineral content (ppm); Zinc, Copper, Iron, Manganese, and Magnesium of *L. dorsi* muscle was determined by an Atomic Absorption Spectrophotometer (GBC 932 AA) according to the method described by Fick *et al* [11].

Color parameters of the *L. dorsi* muscle were measured by a Spectrophotometer (Carry 100 Bio UV-Vis, Varian, Holland) set on the L*, a*, and b* system (CIE lab). The color parameters were analyzed using the color coordinates for CIE lab. Tenderness of meat sampleswere determined by using a texture analyzer (Texture analyzer HD plus, Stable micro systems, UK) equipped with a Warner-Bratzler shear blade.

### Statistical analysis

The data were statistically analyzed by using 2 factor (group and sex) complete randomized design with interaction in SAS 9.3 (2013) software available at Bio-statistical laboratory, College of Veterinary Science, Khanapara received from ICAR, New Delhi under NAIP (Component-1). The *post hoc* test was done by Turkey’s honest significant difference test [12].

## Results and Discussion

Carcass characteristics of the different experimental groups are presented in Table-3. The final body weight of pigs revealed a significant (p<0.01) difference among the groups. Final body weight of pigs in Group E_2_ was higher than those of Group E_1_ and C, whereas not varied with Group E_3_. Furthermore, the final weight of pigs of Group E_1_ was also higher than those recorded for Group C. Sex did not influence on the final body weight of pigs.

**Table-3:** Effect of housing system (deep litter vs. conventional) and feed (fermented vs. conventional) on carcass traits of crossbred Hampshire pigs (values are presented as mean±standard error).

Parameter	Groups				Sex		Probability level
	
	E_1_	E_2_	E_3_	C	M	F	
Initial body weight (kg)	15.08±0.48	15.08±0.51	15.00±0.50	15.04±0.51	15.06±0.32	15.04±0.37	NS
Final body weight (kg)	76.92±0.97^b^	84.25±2.75^a^	80.25±2.08^ab^	74.33±0.74^c^	80.42±1.32	77.46±1.57	**
Pre-slaughter weight (kg)	75.83±1.66^a^	87.67±3.18^b^	81.33±3.26^ab^	74.00±1.15^a^	80.42±2.07	79.00±3.54	**
Hot carcass weight (kg)	54.69±1.26^bc^	64.15±2.21^a^	59.47±2.47^ab^	53.27±0.86^c^	58.23±1.52	57.56±2.00	**
Dressing percentage (%)	72.11±0.09^a^	73.21±0.45^b^	73.11±0.32^b^	71.99±0.29^a^	72.42±40	72.81±0.30	**
Head weight (kg)	6.92±0.11^a^	7.34±0.10^a^	7.19±0.21^a^	6.35±0.22^b^	6.93±0.14	6.97±0.18	**
Heart (kg)	0.26±0.01^c^	0.32±0.01^a^	0.30±0.02^ab^	0.27±0.01^bc^	0.29±0.01	0.29±0.01	*
Kidney (kg)	0.22±0.01	0.23±0.01	0.21±0.01	0.21±0.003	0.22±0.01	0.22±0.01	NS
Liver (kg)	1.39±0.02^b^	1.51±0.04^a^	1.42±0.03^ab^	1.37±0.01^b^	1.42±0.03	1.42±0.02	*
Spleen (kg)	0.21±0.004	0.23±0.01	0.23±0.02	0.20±0.003	0.21±0.04	0.22±0.01	NS
Lung (kg)	0.96±0.04^a^	1.14±0.05^b^	1.08±0.06^ab^	0.96±0.03^a^	1.04±0.03	1.04±0.04	*
Stomach (kg)	0.85±0.02	0.90±0.03	0.90±0.02	0.85±0.03	0.87±0.02	0.88±0.02	NS
Carcass length (cm)	78.33±0.33	80.17±1.17	79.67±1.61	76.17±1.25	78.33±0.79	78.83±1.04	NS
Backfat thickness (cm)	2.06±0.21	2.43±0.19	2.37±0.11	2.06±0.14	2.31±0.11	2.15±0.13	NS
Loin eye area (cm^2^)	36.00±1.61	35.58±0.89	35.67±0.95	34.83±1.35	34.62±0.78	36.42±0.81	NS
Ham (kg)	12.80±0.37^a^	14.85±0.36^b^	14.01±0.30^b^	12.64±0.21^a^	13.56±0.30	13.59±0.42	**
Bacon (kg)	12.61±0.26^a^	15.08±0.55^b^	13.96±0.38^c^	12.33±0.18^a^	13.80±0.43	13.18±0.37	**
Loin (kg)	12.17±0.52^a^	14.60±0.50^b^	13.53±0.83^ab^	12.11±0.18^a^	13.09±0.46	13.12±0.51	*
Boston butt (kg)	8.21±0.18^bc^	9.51±0.51^a^	8.81±0.32^ab^	7.57±0.33^c^	8.68±0.22	8.37±0.39	**
Picnic (kg)	6.17±0.08	7.51±0.70	6.11±0.73	5.89±0.24	6.36±0.36	6.48±0.44	NS
Jowl (kg)	1.75±0.09	1.84±0.06	1.79±0.14	1.75±0.06	1.78±0.05	1.78±0.08	NS
Meat: Bone ratio	4.22±0.19^bc^	4.54±0.12^a^	4.42±0.13^ab^	3.99±0.09^c^	4.45±0.11^A^	4.13±0.11^B^	**
Length of SI (m)	16.90±0.27	17.37±0.13	17.22±0.12	16.73±0.10	17.14±0.16	16.97±0.11	NS
Diameter of SI (mm)	3.11±0.03^a^	3.33±0.08^b^	3.27±0.09^ab^	2.80±0.07^c^	3.12±0.05	3.13±0.10	**
Weight of SI (kg)	1.11±0.02^c^	1.28±0.03^a^	1.19±0.02^b^	1.10±0.02^c^	1.16±0.02	1.18±0.03	**
Length of LI (m)	6.45±0.14	6.82±0.14	6.73±0.11	6.44±0.11	6.74±0.08^A^	6.48±0.10^B^	*
Diameter of LI (mm)	5.92±0.09^bc^	6.16±0.05^ab^	6.20±0.13^a^	5.83±0.07^c^	6.05±0.05	6.00±0.10	*
Weight of LI (kg)	1.06±0.05	1.12±0.07	1.05±0.07	0.87±0.05	1.04±0.05	1.01±0.05	NS

abc Means within a row and group with different superscript significantly differ

AB Means within a row and sex with different superscript significantly differ, E_1_; reared on a conventional housing and fed with a fermented diet. E_2_; reared on a deep litter housing system and fed with a fermented diet. E_3_; reared on a deep litter housing system and fed with a conventional diet and C; reared on a conventional housing system and fed with a conventional diet,

* p<0.05,

** p<0.01, NS=p>0.05, NS=Non-significant

Pre-slaughter weight, hot carcass weight, and dressing percentage of crossbred Hampshire pigs showed a significant (p<0.01) difference among the experimental groups. The pre-slaughter weight of Group E_2_ was significantly higher than Group E_1_ and C, but did not differ from Group E_3_. However, no significant differences were observed among Group E_1,_ E_3_, and C. In regards to hot carcass weight, Group E_2_, was higher than Groups E_1_ and C, but did not differ from Group E_3_, while the difference observed between Group E_3_ and C was found to be significant. Again, the dressing percentage in Group E_2_ and E_3_ were higher than those recorded for Group E_1_ and C. Further, no differences could be observed between Group E_2_ and E_3_ and between Group E_1_ and C. However, sex and interaction of group and sex had no effect. The findings of the present study are in agreement with the report of Paterson *et al*.[13] who observed that pigs housed in the deep litter were 2 kg heavier at slaughter than the pigs housed conventionally. In another study, pigs reared on bedded area showed significantly heavier hot carcass weight (93.2 kg) than those reared on slatted floor (89.6 kg) [14]. Dressing percentage also reported to be 2% higher in pigs finished on deep bedding as compared to pigs finished on slatted floor housing [15]. However, final body weight, carcass weight, and dressing percentage were not affected when pigs fed with dry pelleted feed, fermented feed, and acidified liquid feed [9,16].

In regards to edible offal, a significant difference could be observed in the weight of heart and liver (p<0.05) among the groups while sex had no effect. Heart weight recorded for Group E_2_ was higher than those recorded for Group E_1_ and C but did not differ from E_3_. The value recorded for Group E_3_ also differed from E_1_ but not with Group C. Furthermore, no difference could be seen between Group E_1_ and C. The weight of liver recorded for Group E_2_ was higher than those recorded for Groups E_1_ and C but did not differ from Group E_3_. Analysis also revealed no differences of liver weight among Group E_1_, E_3_, and C. On the other hand; no significant differences were found in relation to the weight of kidney. The weight of expelled blood was also not different among the experimental groups. The results of the present investigation on edible offal of pigs are in close proximity with the findings of Borah [17] who reported that crossbred pigs reared on deep litter and conventional system had weight of liver (1.56±0.05 vs. 1.14±0.05 kg), weight of heart (0.27±0.01 vs. 0.24±0.02 kg), and weight of kidney (0.22±0.01 vs. 0.24±0.02 kg), respectively, and the difference in the weight of liver and heart was statistically significant. In another study, pigs kept in litter bed also had significantly heavier liver and heart weight than those in slatted floor [18].

Among the inedible offal, weight of the head (p<0.01) and lung (p<0.05) differed significantly among the groups. Head weight recorded in Group C was significantly lower than those recorded in Group E_1_, E_2_, and E_3_. However, no differences could be observed among Group E_1_, E_2_, and E_3_. Again, the weight of lung in group E_2_ differed significantly with Group E_1_ and C, but not with Group E_3_ while the insignificant difference was observed between Group E_1_ and C and between Group E_2_ and E_3._ In contrast, no significant difference was noticed in respect of the weight of spleen and stomach among the groups. The result of the present study are in agreement with the findings of Lebret *et al*.[18] who recorded higher average weight of lung, spleen, gastrointestinal tract, and stomach of pigs slaughtered at 110 kg reared on sawdust bedding with free access to an outdoor area compared to fully slatted floor. Lung and head weight was not influenced when pigs were fed with different levels of wet brewer’s grain (WBG) [19].

In relation to carcass measurements *viz*., carcass length, backfat thickness, and loin eye area, no significant (p>0.05) difference was noticed among the experimental groups. Results obtained in the present study corroborated with the earlier findings [20-23]. Moreover, non-significant difference in carcass measurements of pigs between probiotic treated and untreated group was also observed [7].

Wholesale cuts of crossbred Hampshire pigs revealed significant difference in regards to weight of ham (p<0.01), bacon (p<0.01), loin (p<0.05), and boston butt (p<0.01) among the groups while sex had no influence. Weight of ham in Group E_2_ and E_3_ were higher than Group E_1_ and C, while no difference was observed between Group E_2_, E_3_ and Group E_1_, C. Weight of bacon in Group E_2_ was significantly higher than Group E_1_, E_3_, and C. Further, bacon weight recorded for E_3_ was also higher than those recorded for Group E_1_ and C but no difference could be observed between Group E_1_ and C. Again weight of loin in Group E_2_ was recorded higher than those for Group E_1_ and C, however, the differences among Group E_1_, E_3_, and C were not significant. In the case of weight of Boston butt, Group E_2_ differed from Group E_1_ and C but did not differ with Group E_3_. Moreover, Group E_3_ differed from Group C but not with Group E_1_. Furthermore, no difference was observed between Group E_1_ and C. Weight of picnic and jowl were notinfluenced by the treatment groups and also by sex. The average meat: Bone ratio of pigs showed a significant difference among the groups and between sexes (p<0.01). However, the interaction of group and sex had no influence on meat: bone ratio. Higher meat: Bone ratio was recorded in Group E_2_ than those recorded for Group E_1_ and C but not differed with Group E_3_. However, no difference could be observed between Group E_1_ and C while Group E_3_ differed from Group C. The male pigs had higher meat: bone ratio than the female pigs. The results of the present study correspond to the findings of Zhou *et al*. [21] and Pugliese *et al*.[24], who reported higher percentage of loin, ham, shoulder, jowl, and lean cuts in outdoor pigs than those reared indoors. The outdoor pigs also had lower percentage of bone and lean: bone ratio (12.33 vs. 12.49 and 7.08 vs. 7.14, respectively) when compared to indoor pigs [25]. Pigs kept in the deep litter system had higher meat: Bone ratio than those in a conventional system (p<0.01) [17].

Morphometry of intestine revealed a significant difference in respect of diameter (p<0.01) and weight (p<0.01) of small intestine among the groups but the length of the small intestine was not affected. Diameter of small intestine recorded in Group E_2_ was found to be higher than group E_1_ and C, while not varied with E_3_. The value recorded for E_3_ and C were also varied with Group C; however, no difference was noticed between Group E_1_ and E_3_. Weight of small intestine was found to be higher in Group E_2_ than Group E_1_, E_3_, and C. Further, weight of SI in Group E_3_ also higher than Group E_1_ and C, but no difference was observed between Group E_1_ and C. On the other hand, diameter of large intestine showed significant difference among the groups (p<0.05). The diameter of LI was found to be higher in Group E_3_ than E_1_ and C but not differed with E_2_. The values recorded for E_2_ were also varied with those recorded for Group C, however, no difference could be observed between Group E_1_ and C. Length of large intestine in male pigs was found to besignificantly higher than those recorded for the females (p<0.05). Weight of large intestine neither influenced by the experimental groups nor by sexes. When pigs fed high-fiber diets, a significant extension of the Gastrointestinaltract (GI) is observed as a response to the increased volume of the digesta and the increased secretion of digestive fluid [26]. Anincreasing trend of weights of the pig intestine after increasingthe level of WBGdietary supplementation was observed, although the differences were not significant [7].

The chemical composition of *L. dorsi* muscle revealed significant differences in regards to moisture and total ash content among the groups (p<0.05) (Table-4). Moisture percent of pork in Group E_1_ was estimated to be higher than those of Group E_3_ and C, but not varied with Group E_2_. Further, no difference was observed among Group E_2_, E_3_, and C. Total ash content was found to be higher in Group E_3_ and C, than those recorded for E_1_ and E_2_. However, no difference was observed between Group E_1_ and E_2_; and E_3_ and C. Moreover, no significant differences were observed in respect to crude protein and ether extract content. Pork produced in the deep litter had higher crude protein content than that of a conventional system as it was found in a previous study [26]. Pigs reared outdoor also hadhigher crude protein content in meat compared to that of indoor reared pigs [27,28]. In the present study, crude protein level appeared to be slightly higher in pigs reared on deep litter system than that of theconventional system although the difference was not significant. Furthermore, pigs which fed fermented feed had lower ash content in the *L. dorsi* muscle than those fed conventional feed. This finding is not in agreement with the results of a previous study, where the ash content of the muscle was found to be increased as the level of incorporation of fermented feed increased [29].

**Table-4:** Effect of housing system (deep litter vs. conventional) and feed (fermented vs. conventional) on chemical composition, mineral profile, color parameters, and shear force value of *L. dorsi* muscle of crossbred Hampshire pigs of experimental groups (values are presented as mean±standard error).

Parameter	Groups				Sex		Probability level
	
	E_1_	E_2_	E_3_	C	M	F	
Moisture (%)	74.70±0.16^a^	74.32±0.18^ab^	74.08±0.09^b^	74.07±0.09^b^	74.41±0.11	74.18±0.12	*
Crude protein (%)	21.26±0.19	21.63±0.28	21.76±0.27	21.29±0.27	21.42±0.21	21.55±0.15	NS
Ether extract (%)	2.54±0.07	2.45±0.05	2.56±0.06	2.42±0.05	2.55±0.05	2.44±0.02	NS
Total ash (%)	1.00±0.04^a^	1.01±0.39^a^	1.12±0.04^b^	1.16±0.02^b^	1.10±0.03	1.05±0.03	*
Zn (ppm)	2.38±0.46	2.19±0.44	2.59±0.29	2.62±0.18	2.04±0.24	2.85±0.13	NS
Cu (ppm)	1.23±0.65	1.25±0.45	0.49±0.21	0.80±0.29	0.70±0.20	1.19±0.36	NS
Fe (ppm)	2.15±0.26	2.03±0.27	2.28±0.85	2.84±1.03	1.78±0.23	2.87±0.55	NS
Mn (ppm)	0.17±0.09	0.29±0.11	0.34±0.10	0.50±0.38	0.16±0.06	0.49±0.18	NS
Mg (ppm)	3.31±0.32	3.07±0.11	3.76±0.62	2.95±0.34	3.10±0.16	3.45±0.35	NS
L*	2.13±0.02^a^	2.10±0.01^ab^	2.08±0.01^b^	2.09±0.01^b^	2.10±0.01	2.11±0.01	*
a*	0.06±0.01^a^	0.09±0.01^b^	0.04±0.005^a^	0.05±0.01^a^	0.06±0.01	0.06±0.01	*
b*	0.79±0.01	0.75±0.01	0.77±0.01	0.76±0.01	0.77±0.01	0.77±0.01	NS
Shear force value (kg)	0.0079±0.002^a^	0.0077±0.002^a^	0.011±0.002^a^	0.0164±0.001^b^	0.0113±0.002	0.0103±0.001	**

ab means within a row and group with different superscript significantly differ, ^ab^means within a row and sex with different superscript significantly differ, E_1_; reared on a conventional housing and fed with a fermented diet, E_2_; reared on a deep litter housing system and fed with a fermented diet, E_3_; reared on a deep litter housing system and fed with a conventional diet and, C; reared on a conventional housing system and fed with a conventional diet,

* p<0.05,

** p<0.01, NS=p>0.05, NS=Non-significant

The mineral concentration *viz*., Zn, Cu, Fe, Mn, and Mg concentration of *L. dorsi* muscle of pigs did not vary among the groups, between sex and interaction of group and sex (Table-4). However, an earlier report of Gentry *et al*. [22] showed that pigs reared outdoor had higher plasma iron content than pigs reared indoors.

Values of color parameters in the *L. dorsi* muscle were significantly different among groups (p<0.05). The L* value was found to be higher in Group E_1_ than those of Group E_3_ and C but did not differ from Group E_2_. The difference between Group E_3_ and C also not varied. The value of parameter a* in Group E_2_ was appeared to be higher than the other experimental groups. However, the differences among Group E_1_, E_3_, and C were not significant. For the parameter b* the differences among the groups were not significant. The results of the present study are in agreement with the findings of Dimatteo *et al* [30] and Trezona *et al* [31] who reported that color parameters for L*, a*, b* of the quadriceps *femoris* and *L. dorsi* muscle were significantly (p<0.05) higher in meat of pigs reared in straw bedded than concrete floor. On the other hand, pigs which fed fermented food waste feed had higher L* and lower a* value in loin muscle that those fed control ration [9].

In regards to the textural properties of meat, shear force value of *L. dorsi* muscle showed a significant difference (p<0.01) among the experimental groups. Pork produced in Group C was found to be less tender than the other experimental groups. However, no significant differences could be observed among Group E_1_, E_2_, and E_3_. The present findings corroborated with reports made by Beattie *et al*. [28] who found tender pork produced by deep litter pigs than those from the slatted floor. In another investigation, it was observed that shear force value decreased by 11.76% in pork from pigs reared in deep fermented litter than conventionally kept pigs [20]. A possible explanation for this difference is that enriched pigs had higher levels of intramuscular fat, which are associated with improved tenderness and water holding capacity in pork [32]. On the other hand, pigs fed with fermented feed produced tender pork than those fed control feed [8] which is in agreement with the results of the present study.

## Conclusion

From the present study, it may be concluded that crossbred Hampshire pigs Group E_2_ (reared on a deep litter housing system and fed with a fermented diet) showed increased slaughter weight and hot carcass weight than the other experimental groups. Dressing percentage calculated for E_2_ Group was higher by 1.1% over E_1_ (reared on a conventional housing and fed with a fermented diet), 0.1% over Group E_3_ (reared on a deep litter housing system and fed with a conventional diet), and 1.22% over Group C (reared on a conventional housing system and fed with a conventional diet). The carcass measurements were affected neither by rearing system nor by feeding fermented feed. Crossbred pigs of E_2_ Group showed improved wholesale cuts than the other experimental groups. Finally, crossbred pigs reared in Group E_1_ produced brighter meat while those of E_2_ Group produced more red meat. Meat of E_2_ Group was found to be softer than that of other experimental groups. Therefore, deep litter housing and feeding of fermented feed may be suggested for improvement in carcass characteristics and meat quality of crossbred Hampshire pigs.

## Author’s Contributions

This experiment was a Ph.D. Research study of the first author MR. JRB acted as major advisor and contributed immensely right from the start to end of the experiment. AKS contributed in statistical analysis part. RR and AB contributed valuable suggestions during the experiment. All authors read and approved the final manuscript.
